# 4-Phenethyl-1-Propargylpiperidine-Derived Dual Inhibitors of Butyrylcholinesterase and Monoamine Oxidase B

**DOI:** 10.3390/molecules26144118

**Published:** 2021-07-06

**Authors:** Tjaša Mazej, Damijan Knez, Anže Meden, Stanislav Gobec, Matej Sova

**Affiliations:** Faculty of Pharmacy, University of Ljubljana, Aškerčeva Cesta 7, SI-1000 Ljubljana, Slovenia; tjasa.mazej@gmail.com (T.M.); damijan.knez@ffa.uni-lj.si (D.K.); anze.meden@ffa.uni-lj.si (A.M.); stanislav.gobec@ffa.uni-lj.si (S.G.)

**Keywords:** Alzheimer’s disease, butyrylcholinesterase, monoamine oxidase B, inhibitors, multi-target-directed ligands, multifunctional ligands

## Abstract

The multi-target-directed ligands (MTDLs) strategy is encouraged for the development of novel modulators targeting multiple pathways in the neurodegenerative cascade typical for Alzheimer’s disease (AD). Based on the structure of an in-house irreversible monoamine oxidase B (MAO-B) inhibitor, we aimed to introduce a carbamate moiety on the aromatic ring to impart cholinesterase (ChE) inhibition, and to furnish multifunctional ligands targeting two enzymes that are intricately involved in AD pathobiology. In this study, we synthesized three dual hMAO-B/hBChE inhibitors **13**–**15**, with compound **15** exhibiting balanced, low micromolar inhibition of hMAO-B (IC_50 _of 4.3 µM) and hBChE (IC_50_ of 8.5 µM). The docking studies and time-dependent inhibition of hBChE confirmed the initial expectation that the introduced carbamate moiety is responsible for covalent inhibition. Therefore, dual-acting compound **15** represents an excellent starting point for further optimization of balanced MTDLs

## 1. Introduction

Alzheimer’s disease (AD) is a progressive neurodegenerative brain disorder characterized by memory deterioration, behavioral changes, and impaired cognitive functions [1,2]. Although the exact aetiology of AD remains to be clarified, the pathogenesis is considered to be multifactorial [1,3]. The proposed pathophysiological mechanisms include amyloid β deposition [4], protein tau aggregation [5], increased oxidative stress [6], imbalance of metal ions [7], mitochondrial dysfunction [6,8], and neuronal and synaptic loss in the central nervous system [9]. Neurodegeneration includes alterations in neurons, microglia, and astroglia, which altogether drive the insidious progression of AD long before the clinical symptoms are detected [2]. Neuroinflammation intertwined with amyloid β and tau pathology drives the progressive loss of neuronal tissue. Misfolded proteins, i.e., amyloid β and tau, bind to pattern recognition receptors on astrocytes and microglia, and trigger an innate immune response characterized by a release of pro-inflammatory mediators, which further exacerbates the loss of neurons [10,11]. The neurodegenerative processes predominantly affect the cholinergic system, and result in lowered levels of acetylcholine (ACh) that impedes learning processes and memory formation [12,13]. Inhibition of cholinesterases (ChEs), which catalyze the breakdown of ACh, increases the local concentration of ACh and improves cholinergic transmission [14]. With the progression of AD, the activity of acetylcholinesterase (AChE) decreases, whereas the activity of butyrylcholinesterase (BChE) is compensatorily increased [14,15,16]. Additional potential for the mitigation of AD progress arises through the inhibition of monoamine oxidases (MAOs), which during the oxidative deamination reaction produce mediators of oxidative stress, such as hydrogen peroxide, aldehydes, and ammonia. Among the two MAO isoforms, monoamine oxidase A (MAO-A) and B (MAO-B), the latter is implicated in neurodegeneration, as the activity of MAO-B is significantly increased in the brains of AD patients [17,18].

Due to the pathophysiological complexity of AD, the multi-target-directed ligand (MTDL) design strategies are used for the development of the molecules capable of simultaneously modulating multiple pathways in the neurodegenerative cascade [19,20]. Several pathophysiological mechanisms of AD have been discovered and studied in recent decades. Nonetheless, improving cholinergic function remains the only option for the mitigation of Alzheimer’s disease symptoms [13,21,22]. Improving cholinergic neurotransmission and addressing the underlying causes of neurodegeneration in a single ligand is therefore a reasonable path followed in drug development [2,23].

Following the MTDL approach, ladostigil was designed by introducing the carbamate moiety present in the anti-ChE drug rivastigmine into the structure of the irreversible MAO-B inhibitor rasagiline [24,25]. This dual-acting AChE/BChE and brain-selective MAO-A/MAO-B inhibitor, with additional neuroprotective properties due to the presence of propargyl moiety, reached phase II clinical trials in patients with mild cognitive impairment. Despite having a good safety profile, ladostigil did not halt the disease progression to full dementia [26,27]. Numerous studies followed the same approach and developed other propargyl-based dual ChE/MAO inhibitors, which have been extensively reviewed elsewhere [26,28].

**Figure 1:**
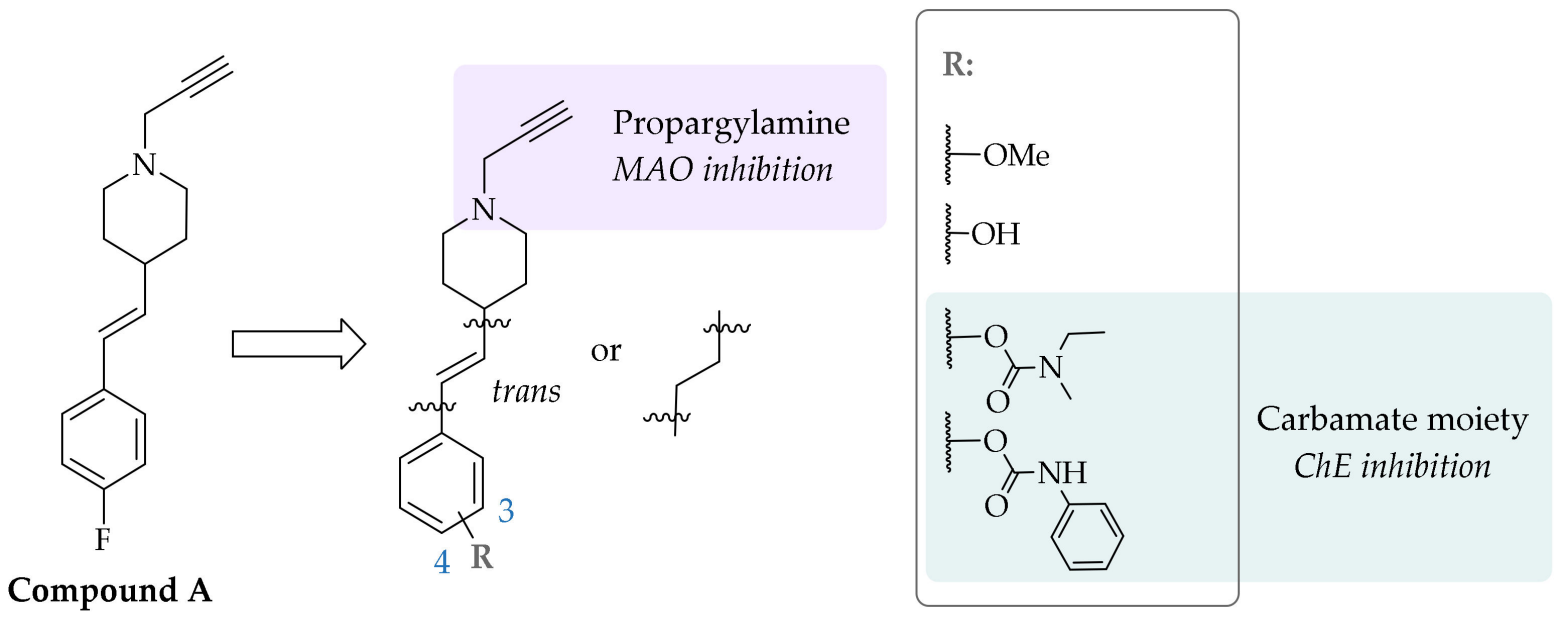
Starting compound **A** and designed *N*-propargylpiperidine carbamates.

An in-house library screening campaign identified compound **A** (Figure 1) as a potent and irreversible MAO-B inhibitor that bound covalently to the *N*5 atom of the flavine adenine dinucleotide (FAD) cofactor of MAO-B, as was revealed by the resolved crystal complex structure [29]. An extensive series of analogues with 1-propargyl-4-styrylpiperidine scaffold was synthesized to explore the structure–activity relationships (SARs). The substitution patterns on the phenyl ring were varied to enhance the selectivity and potency of MAO-A or MAO-B inhibition. In the study presented herein, we introduced a carbamate moiety on the aromatic ring to gain ChE inhibition as well, through the carbamoylation of catalytic Ser198. This approach led to the development of multifunctional ligands targeting two enzymes that are intricately involved in AD pathobiology (Figure 1) [30].

## 2. Results and Discussion

### 2.1. Chemistry

**Scheme 1:**
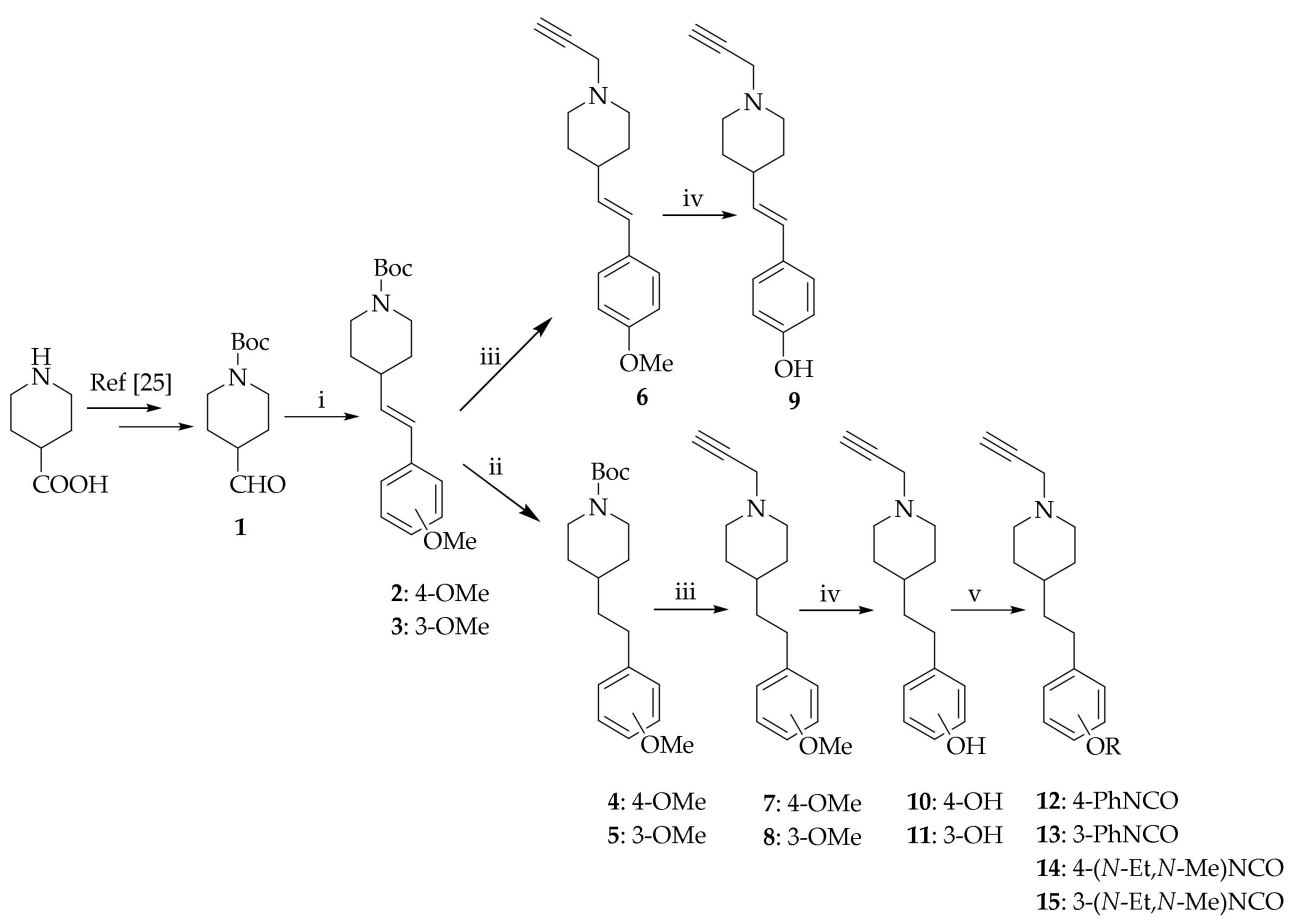
Reagents and conditions: (i) (3- or 4-methoxybenzyl)triphenylphosphonium chloride, sodium bis(trimethylsilyl)amide (NaHMDS), THF, rt, overnight (overall yield: **2**, 60%; **3**, 63%); (ii) H_2_, Pd/C, EtOH, rt, overnight (yield: quantitative); (iii) (1) conc. HCl, EtOH, 80 °C, 2 h (yield: quantitative); (2) propargyl bromide (80% in toluene), K_2_CO_3_, Cs_2_CO_3_, CH_3_CN, 0 °C to rt, 6−24 h (yield: 24%−70%); (iv) BBr_3_, toluene, –80 °C 15 min, 0 °C 30 min, rt 90 min (yields: 13%−30%); (v) for compounds **12** and **13**: 4-dimethylaminopyridine (DMAP), Et_3_N, phenyl isocyanate (PhNCO), CH_2_Cl_2_, rt, overnight (yield: **12**, 30%, **13**, 61%); for compounds **14** and **15**: *N*-ethyl-*N*-methylcarbamoyl chloride, pyridine, rt, 1.5 h (yield: **14**, 83%, **15**, 38%).

The synthetic procedures are presented in Scheme 1. The starting piperidine-4-carboxylic acid was Boc-protected and subsequently converted into aldehyde **1** via the Weinreb amide as described previously [29]. The aldehyde was then subjected to the Wittig reaction with 3- or 4-methoxybenzyl triphenylphosphonium ylide to form the mixture of *E*/*Z*-alkenes **2** and **3**, which were (partially) separated by column chromatography into *E-*isomer and a mixture of *E*/*Z*-isomers. The latter was subjected to catalytic hydrogenation to obtain the corresponding saturated analogues **4** and **5**. Following acidolysis with hydrochloric acid, the secondary amine was alkylated with propargyl bromide to obtain the final compounds **6**–**8**. The methoxy substituents were removed using BBr_3_ to form phenols, which were then carbamoylated with phenyl isocyanate or *N*-ethyl-*N*-methylcarbamoyl chloride to yield carbamates **12**–**15**.

### 2.2. Monoamine Oxidase and Cholinesterase Inhibitory Potencies

Inhibitory potencies against human (h)AChE and hBChE were determined using the Ellman method [31,32] and inhibitory potencies against hMAO-A and hMAO-B were determined with horseradish peroxidase (HRP)-Amplex Red coupled assay [32,33]. The results are reported in Table 1, where inhibitory potencies are expressed as residual activities (RA in percentages at 100 µM compound concentration) or IC_50_ values.

**Figure 2:**
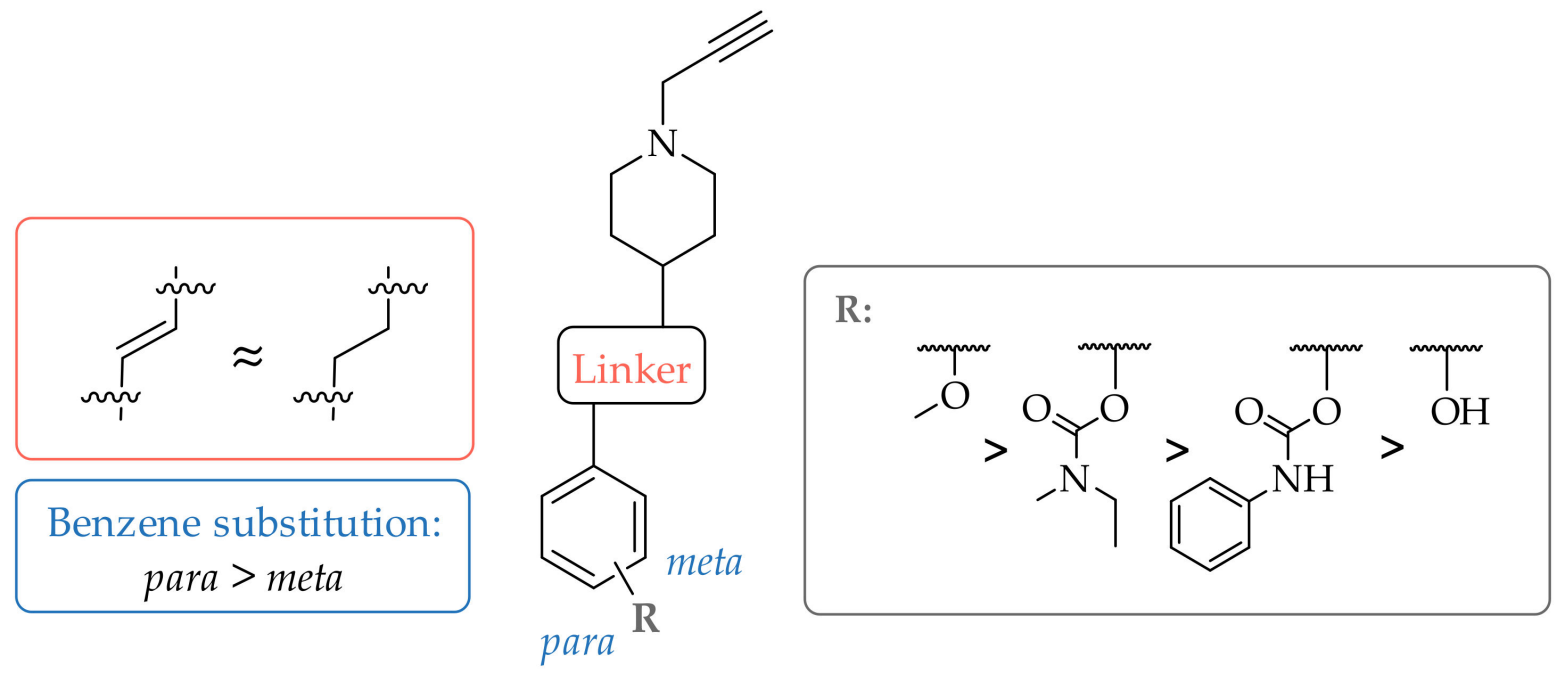
Structure activity relationships for hMAO-B inhibition.

**Figure 3:**
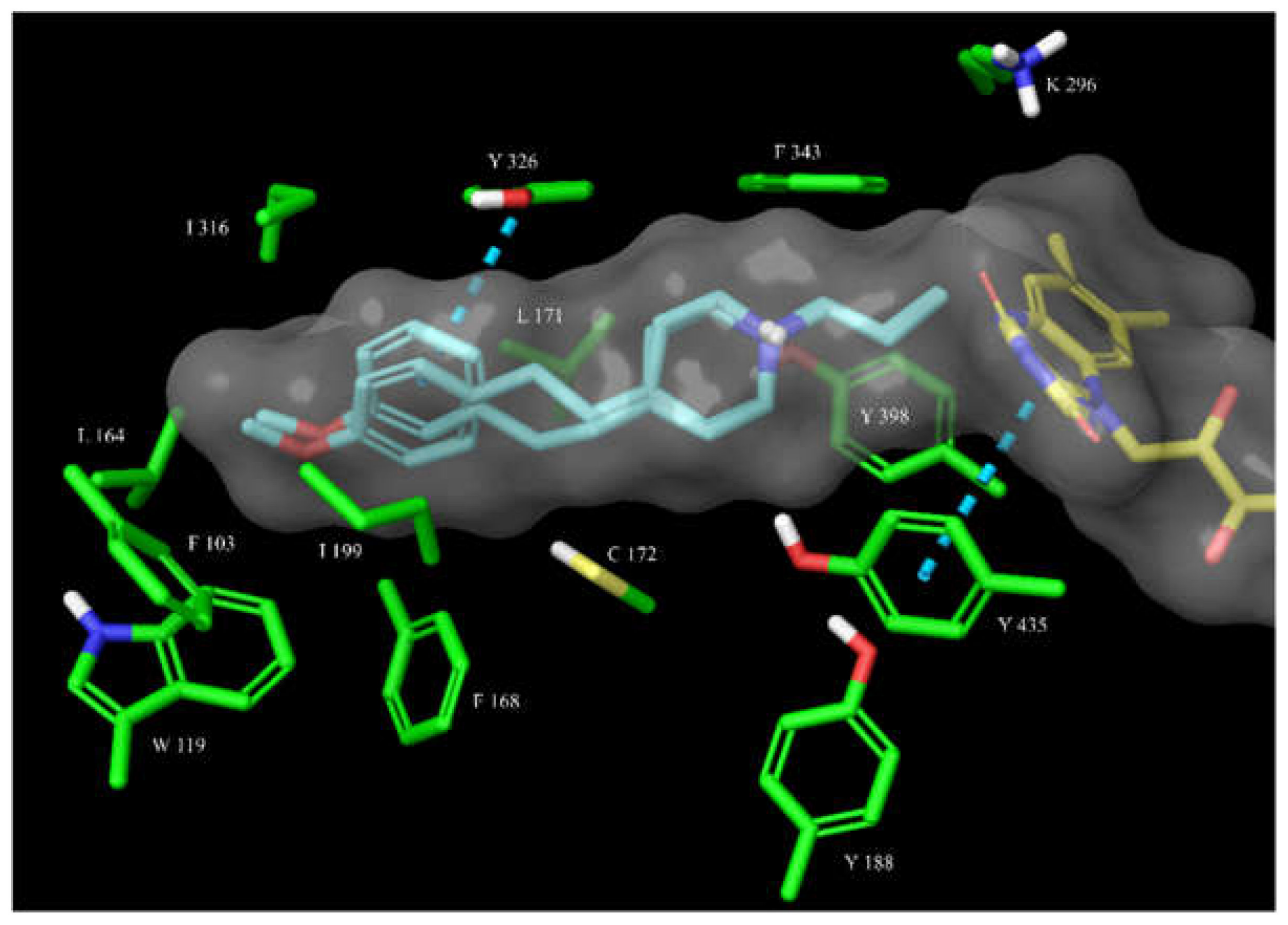
The overlay of docked covalently bound **7** and **8** (shown as cyan sticks) in the hMAO-B active site. The active site is shown as surface with the relevant amino acid residues shown as green sticks and the flavine adenine dinucleotide (FAD) cofactor as yellow sticks. The π-π interactions are represented by blue dashed lines. The conformations of both ligands are very alike.

All compounds inhibited hMAO-B in nano- to micromolar range, whereas only **9** and **10** inhibited hMAO-A with IC_50_ values of 15.5 ± 0.9 µM and 34.0 ± 5.8 µM, respectively (Table 1). Relevant findings regarding SARs for hMAO-B inhibition are summarized in Figure 2. The most potent hMAO-B inhibitor was compound **6**, a 1,4-disubstituted *trans*-styrene derivative and methoxy substituent (IC_50_ = 72.3 ± 7.5 nM). The reduction of the double bond had no significant effect on hMAO-B inhibition, e.g., *trans*-ethylene analogue **6** with IC_50_ of 72.3 ± 7.5 nM vs. saturated analogue **7** with IC_50_ of 93.8 ± 4.1 nM. Comparing the *meta* and *para* substitution of the phenyl ring, at least a 2.5-fold superior inhibition of hMAO-B was observed for the *para* substituted *trans*-styrene counterparts (Figure 3). The hydrophobicity and the size of the phenyl ring substituent also influenced hMAO-B inhibition. In general, increasing hydrophobicity and size improved inhibition of hMAO-B (–OCH_3_ ≫ –OH), with the exception of voluminous carbamate derivatives. These findings are in accordance with the previously described SARs of analogues [29], and are in good agreement with hMAO-B structural characteristics [34]. The narrow and curved entrance to the active site of hMAO-B hinders entrance of relatively wider *meta* substituted analogues, thus explaining their lower inhibitory potencies compared to the *para* substituted counterparts [29].

**Figure 4:**
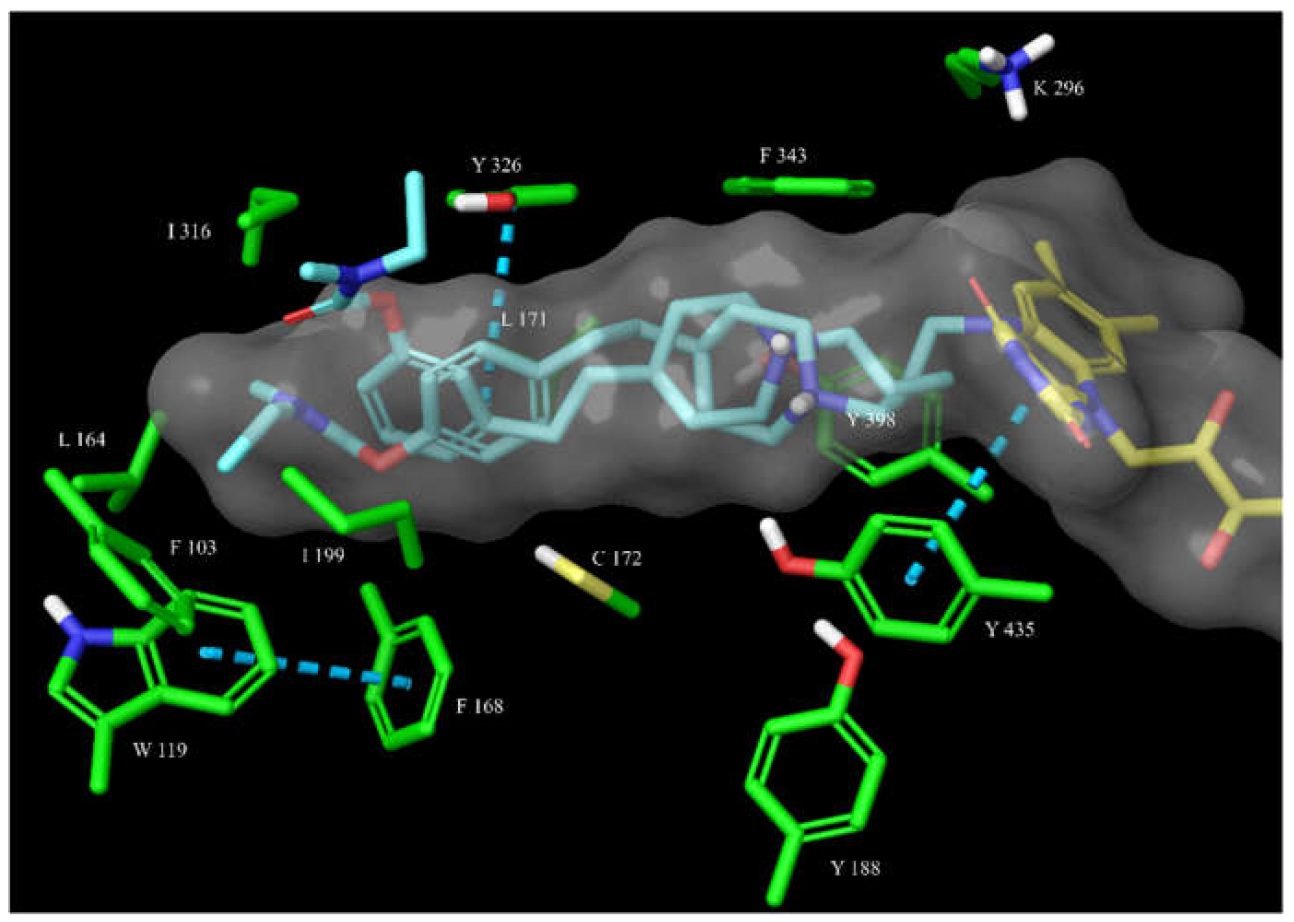
The overlay of docked covalently bound **14** and **15** (shown as cyan sticks) in the hMAO-B active site. The active site is shown as surface with the relevant amino acid residues shown as green sticks and the FAD cofactor as yellow sticks. The π-π interactions are represented by blue dashed lines. The bound *para*-substituted **14** better fits into the active site cavity, forming a π-π interaction with Tyr326, while the protonated nitrogen forms a cation-π interaction with Tyr398 and a hydrogen bond with Gln206.

Additionally, the bulky carbamate substituents can act as a steric obstacle, limiting effective binding in the hMAO-B active site cavity. The presence of the hydrophilic phenol also impedes binding to the highly hydrophobic active site [17,34] contributing to low inhibitory potencies of phenol derivatives. The most probable mechanism of inhibition is the formation of a covalent adduct between the FAD cofactor and the propargylamine moiety present in all compounds. The formation of this bond was confirmed for the analogue series of compounds with crystallographic structural studies [29], and is also tentatively assigned to the *N*-ethyl-*N*-methyl carbamate **14** judging by time-dependent and irreversible inhibition, and covalent docking (Figure 4).

**Figure 5:**
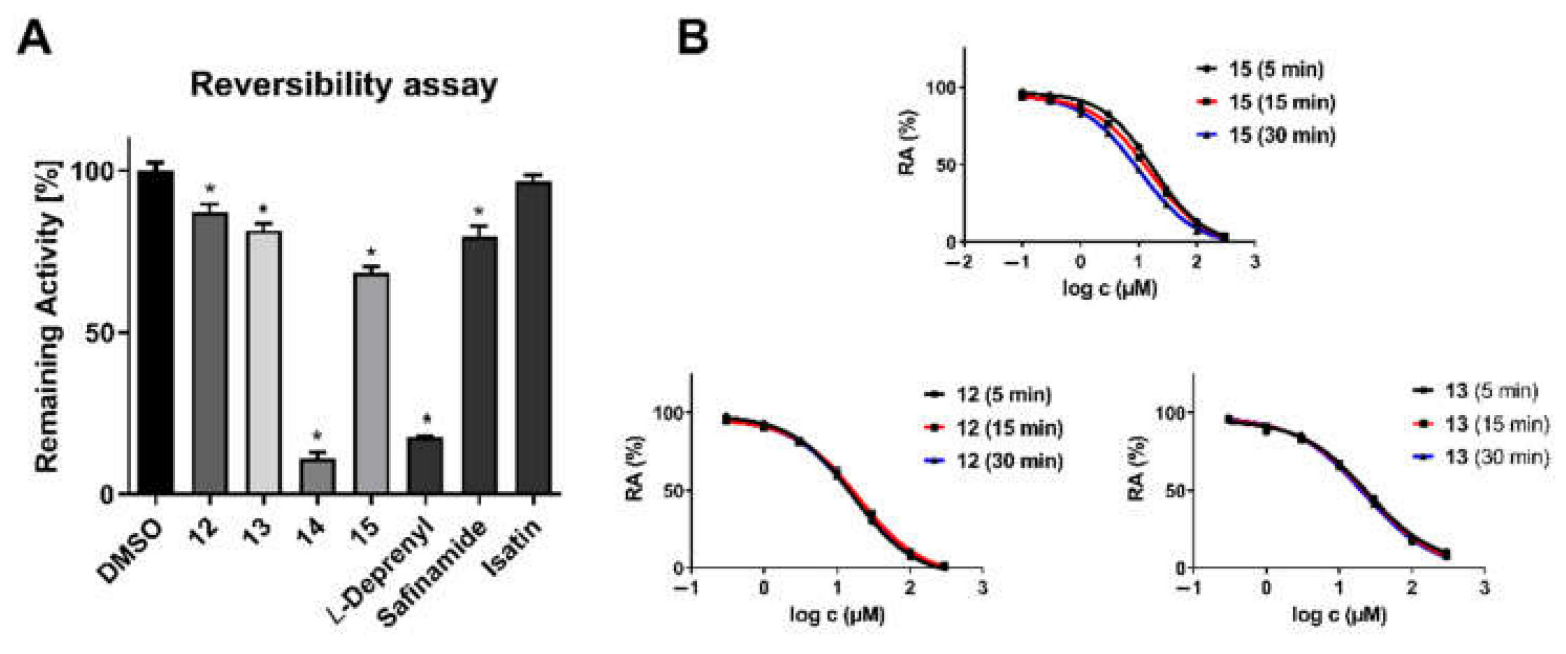
(**A**) A 100-fold dilution assay for hMAO-B inhibitors **12**–**14**. *L*-Deprenyl was used as an irreversible inhibitor control; safinamide and isatin were used as reversible inhibitor controls. Data are mean values ± SEM (*n* = 2; each in quadruplicate): ∗, *p* < 0.05 (one-way ANOVA, with Bonferroni correction, followed by *t*-tests). (**B**) The shifted IC_50_ curve for compound **14** at various pre-incubation times suggests time-dependent hMAO-B inhibition. The absence of IC_50_ shift for compounds **12** and **13** suggests reversible inhibition.

**Figure 6:**
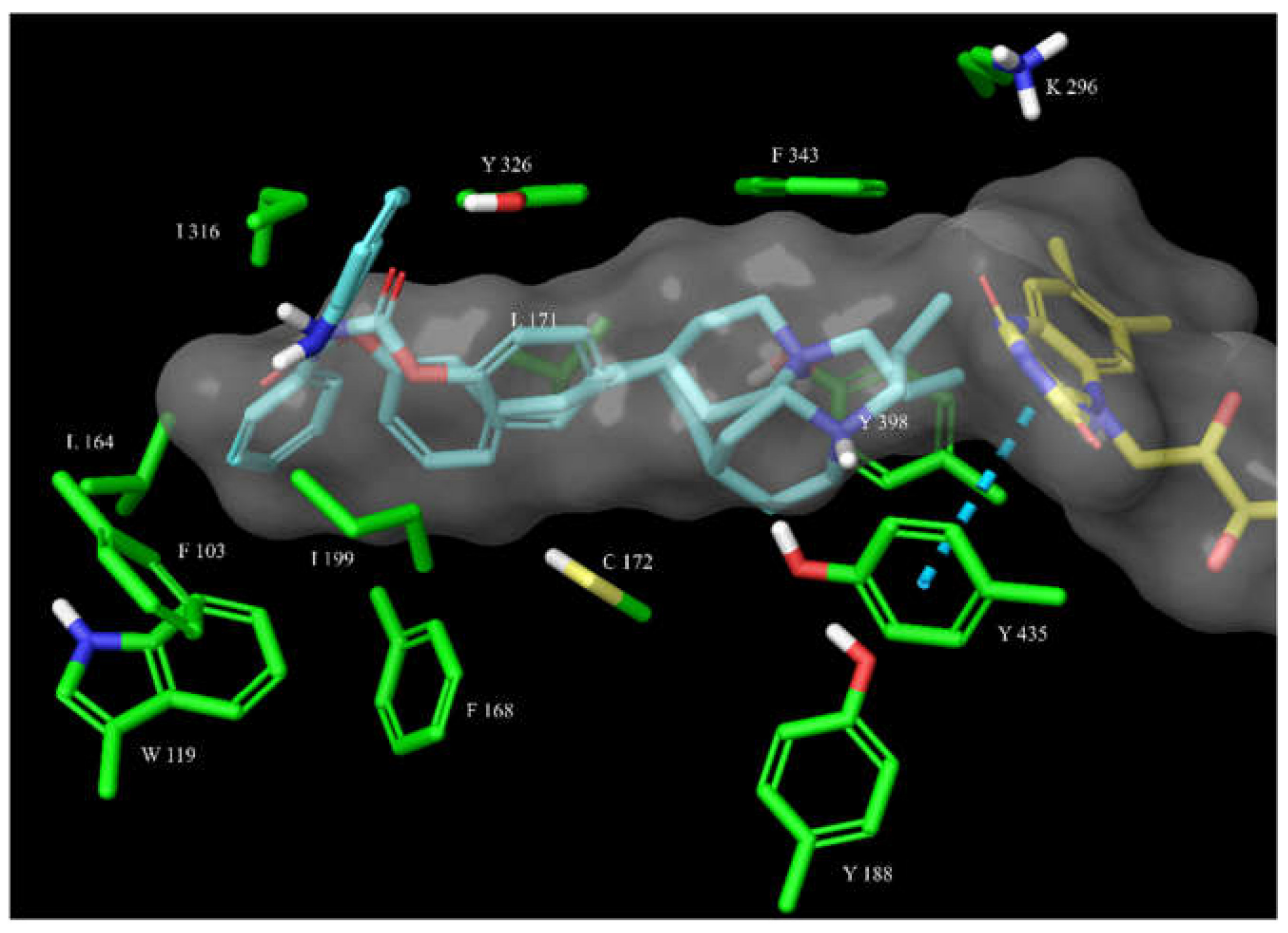
The overlay of docked covalently bound **12** and **13** (shown as cyan sticks) in the hMAO-B active site. The active site is shown as surface with the relevant amino acid residues shown as green sticks and the FAD cofactor as yellow sticks. The π-π interactions are represented by a blue dashed line. Both inhibitors display steric discomplementarity with the extended, flat active site cavity.

Interestingly, the bulkier phenyl carbamates **12** and **13** did not exhibit time-dependent inhibition (Figure 5). As revealed by the 100-fold dilution assay, the inhibition was indeed reversible, which suggests that the voluminous substituents prevented the propargylamine moiety from reaching the FAD cofactor and allowing the formation of a covalent bond. This was also suggested by covalent docking studies (Figure 6).

**Figure 7:**
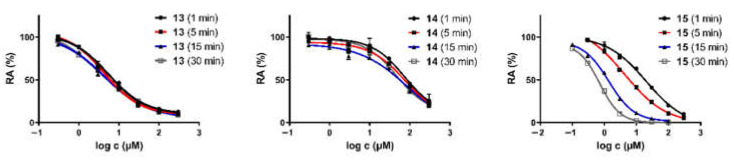
The IC_50_ shift curves for compounds **13**–**15** during hBChE inhibition.

**Figure 8:**
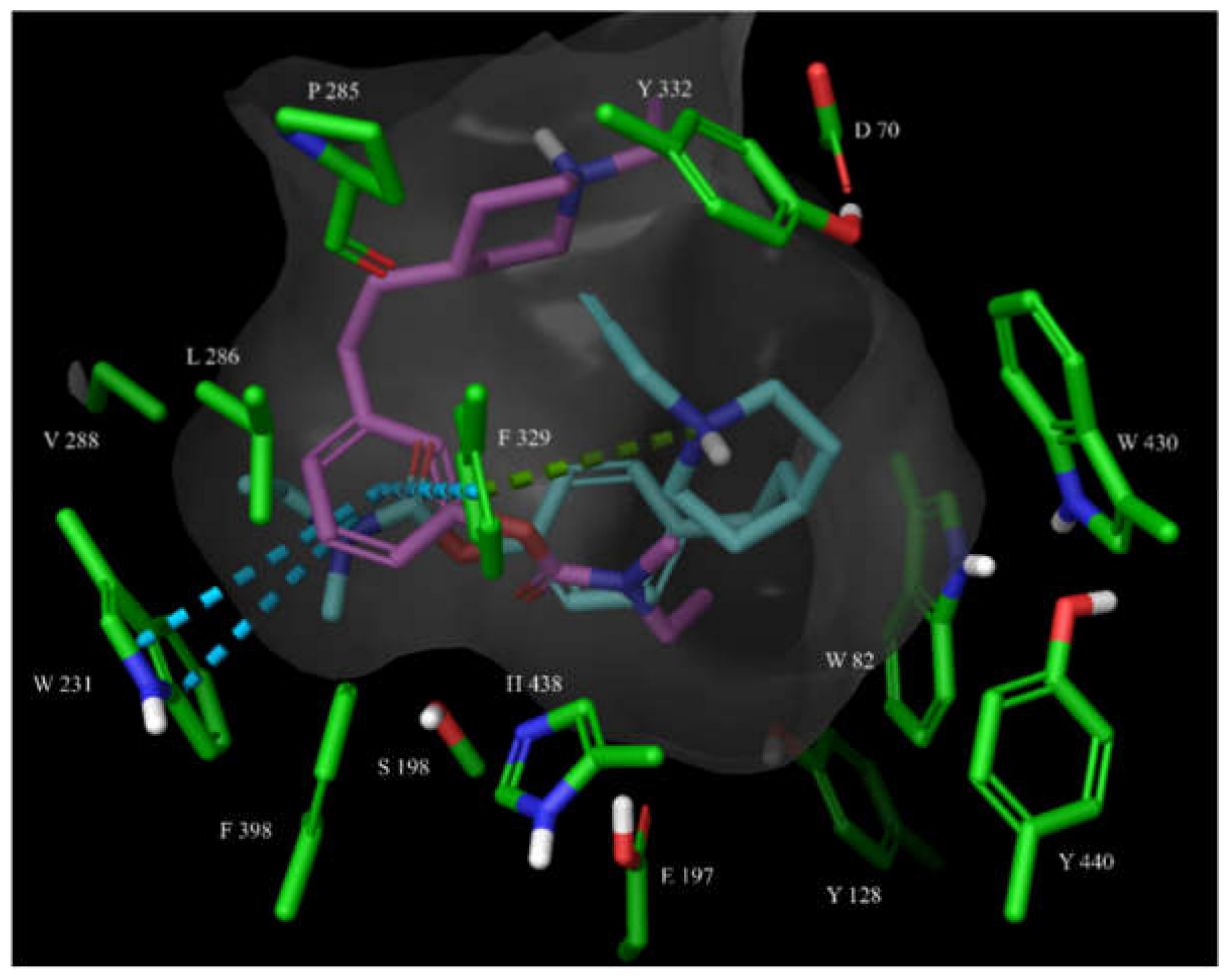
The overlay of docked **14** and **15** (shown as magenta and teal sticks, respectively) in the hBChE active site. The active site is shown as surface with the relevant amino acid residues shown as green sticks. The π-π interactions (shown as blue dashed lines) of **15** with Trp231 and Phe329 are a prerequisite for a successful covalent bond formation, as we have noticed in another series of carbamate hBChE inhibitors (unpublished results).

**Figure 9:**
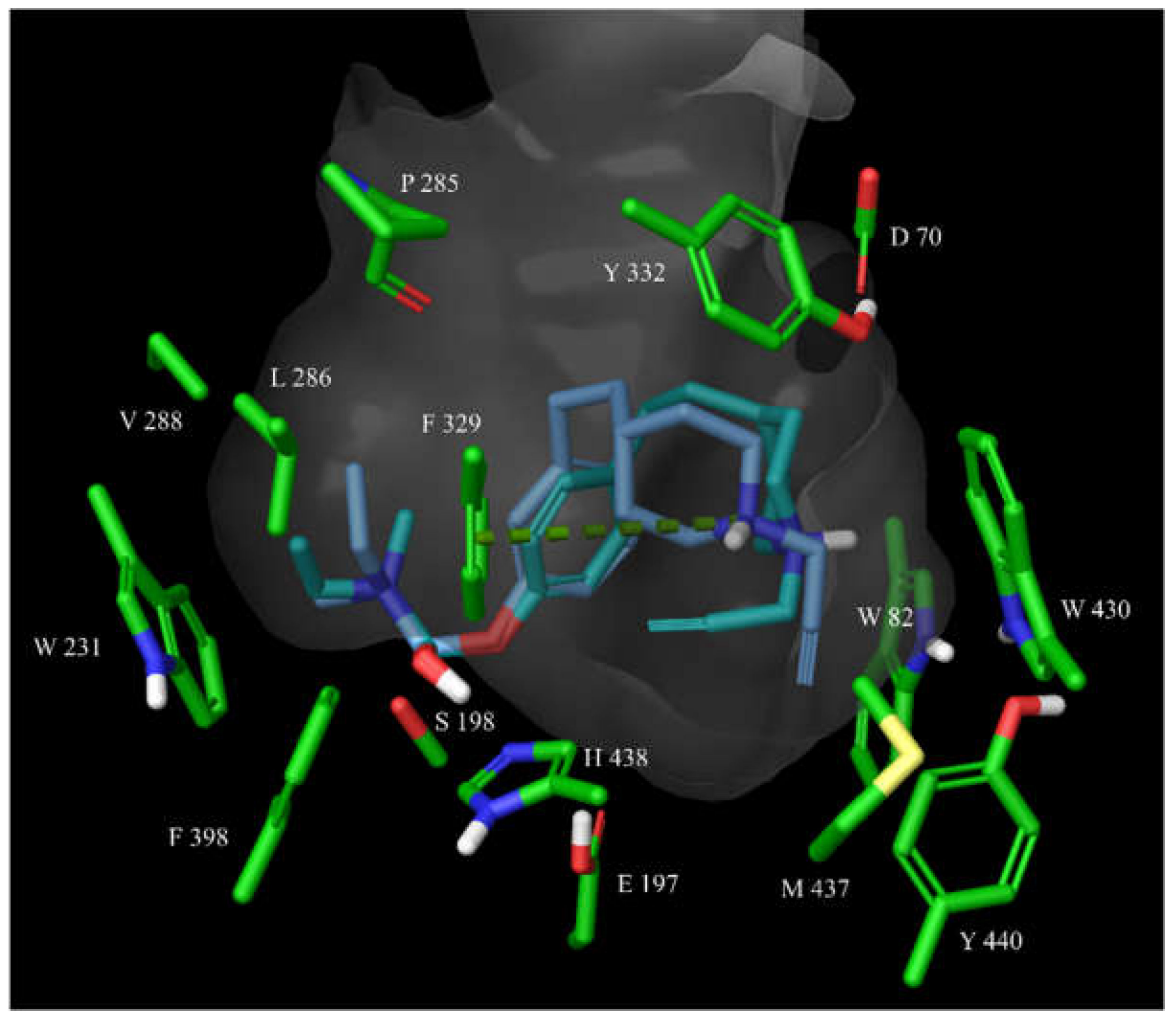
The overlay of docked, covalently bound **14** and **15** (shown as magenta and teal sticks, respectively) in the hBChE active site. The active site is shown as surface with the relevant amino acid residues shown as green sticks. The cation-π interactions of **15** with Phe329 are shown as a green dashed line.

An in vitro assay showed that none of the compounds inhibited hAChE (IC_50_ > 100 µM), while derivatives **13**–**15** inhibited hBChE in the micromolar range. Selective hBChE inhibition can be explained by comparing the active site size of both ChEs, as the 200 Å^3^-larger hBChE active site is easily accessible, whereas the smaller hAChE active site is sterically hindered by numerous aromatic amino acid residues [35]. The presence of carbamate moiety was essential for hBChE inhibition; however, not only due to covalent binding. Importantly, *meta*-positioned carbamates **13** and **15** were more potent hBChE inhibitors in comparison to their *para* congeners **12** and **14**, respectively. Higher inhibitory potencies of *meta* derivatives could be explained by the superior binding of more branched *meta* analogues to the large hBChE cavity compared to the 1,4-derivatives (e.g., *para* **14**, IC_50_ = 75.5 ± 8.4 µM vs. *meta* **15**, IC_50_ = 4.3 ± 0.8 µM). The docking results support this rationale; however, the precise binding pose of the compounds in the active site should be unambiguously clarified with crystallographic studies [30]. The time-dependent inhibition of hBChE by *N*-ethyl-*N*-methyl carbamate **15,** as seen in Figure 7, indicates carbamoylation of catalytic Ser198, which is also supported by the non-covalent and covalent docking studies (Figure 8 and Figure 9, respectively). On the other hand, IC_50_ shift was absent for compounds **13** and **14** (Figure 7), making them reversible inhibitors.

## 3. Materials and Methods

### 3.1. Chemistry

#### 3.1.1. General Information

The reagents and solvents used were obtained from commercial sources and were used as provided. Anhydrous tetrahydrofuran (THF) was distilled from sodium-benzophenone and anhydrous dichloromethane (CH_2_Cl_2_) was prepared by distillation from calcium hydride. Analytical thin-layer chromatography was performed on silica-coated aluminum plates (60 F254, 0.20 mm; Merck, Darmstadt, Germany). Flash column chromatography was performed on silica gel 60 (particle size 0.040−0.063 mm, Merck, Darmstadt, Germany). ^1^H-NMR and ^13^C spectra were recorded at 400 and 100 MHz, respectively, on a Bruker Avance III NMR spectrometer (Bruker, MA, USA) at 295 K. The chemical shifts (*δ*) are reported in ppm and are referenced to the residual undeuterated solvent peak in ^1^H or deuterated solvent peak in ^13^C spectra, respectively. HRMS measurements were performed on an Exactive^TM^ Plus Orbitrap Mass Spectrometer (Thermo Scientific^TM^, Waltham, MA, USA). Analytical reversed-phase HPLC analyses were performed on a modular system (Thermo Scientific Dionex UltiMate 3000; Thermo Fisher Scientific Inc., MA, USA) using a ZORBAX Extend C18 column (4.6 × 150 mm, 3.5 μm), thermostated at 50 °C with a sample concentration of 0.1 mg/mL (100% MeCN), a flow rate of 1.0 mL/min, and a detection wavelength set to 220 nm. Mobile phase: A (0.1% TFA (*v*/*v*) in water) and B (MeCN), using gradient elution: 0−12 min, 10%−90% B; 12−14 min, 90% B; 14−15 min, 90%−10% B. The purity of the tested compounds was ≥95%, as determined by HPLC.

#### 3.1.2. General Experimental Procedures

##### General Procedure A: Synthesis of Phosphonium Salts

The appropriate benzyl halide (4-methoxybenzyl chloride or 3-methoxybenzyl chloride) (1.0 equiv.) was dissolved in MeCN (25−50 mL), and triphenylphosphine (1.0 equiv.) was added. The reaction mixture was stirred at 85 °C for 24 h. The solvent was evaporated, and CH_2_Cl_2_ (10 mL) and Et_2_O (50 mL) were added to the residue. The precipitated solid was filtered off, washed with Et_2_O (20 mL), and left on the benchtop to dry overnight. The crude product was used in the following reaction step without further purification.

##### General Procedure B: Wittig Reaction

The appropriate phosphonium ylide (1.0 equiv.) was dissolved in anhydrous THF (50 mL) under Ar and NaHMDS (38% solution in THF, ca. 1.9 mol/L) (1.2 equiv.) was added to the resulting mixture. Compound **1** (1.0 equiv.) was dissolved in anhydrous THF (10 mL) and added dropwise to the reaction mixture, which was then stirred at room temperature for 12 h. The solvent was evaporated and EtOAc (50 mL) and saturated aqueous NaHCO_3_ solution (50 mL) were added to the residue. The resulting phases were separated and the aqueous phase was additionally extracted with EtOAc (2 × 50 mL). Combined organic phases were washed with saturated brine (100 mL) and dried over anhydrous Na_2_SO_4_. The solvent was evaporated and the crude product was purified by column chromatography (EtOAc/*n*-hexane = 1/9 (*v*/*v*)).

##### General Procedure C: Reduction of Double Bond

The alkene (1.0 equiv.) was dissolved in EtOH (50 mL) and purged with Ar for 10 min. A catalytic amount of Pd/C (10% on carbon, 10−20% (*w*/*w*) calculated on starting material) was added and the resulting suspension was purged with H_2_ for 20 min. The reaction was then stirred for 12 h at room temperature under H_2_ (balloon). The catalyst was removed by filtration through Celite and the solvent was evaporated.

##### General Procedure D: Boc-Protection Removal

Boc-protected starting compound (1.0 equiv.) was dissolved in EtOH (30 mL) and concentrated HCl (10.0 equiv.) was added at room temperature. The reaction mixture was stirred at 80 °C for 2 h and 1 h at room temperature. The solvent was evaporated and the residue was dried overnight at 50 °C. The resulting crude product was used in the following reaction step without further purification.

##### General Procedure E: Alkylation of Secondary Amine

Amine hydrochloride intermediate (1.0 equiv.) was dissolved in MeCN under Ar atmosphere. After the addition of K_2_CO_3_ (3.0 equiv.) and Cs_2_CO_3_ (1.0 equiv.), the reaction mixture was cooled to 0 °C and propargyl bromide (80% solution in toluene, 1.2 equiv.) was added dropwise. The reaction was stirred at room temperature from 6–24 h under the Ar atmosphere, protected from the light. The solvent was evaporated, and residue was dissolved in CH_2_Cl_2_ (100 mL). The organic phase was transferred into the separating funnel and washed with saturated aqueous NaHCO_3_ solution (50 mL), saturated brine (50 mL), and then dried over anhydrous Na_2_SO_4_. The solvent was evaporated, and the crude product was purified by column chromatography (EtOAc/*n*-hexane = 1/1 (*v*/*v*)).

##### General Procedure F: Demethylation

The methoxy substituted derivative (1.0 equiv.) was dissolved in anhydrous toluene (25–50 mL), purged under a stream of Ar for 10 min, and cooled to −80 °C. BBr_3_ (1 M solution in CH_2_Cl_2_, 3.0 equiv.) was added dropwise and the reaction was stirred for 15 min at –80 °C, followed by stirring for 30 min at 0 °C and 90 min at room temperature under Ar. The reaction was stopped by the gradual addition of saturated aqueous NaHCO_3_ solution (20 mL) with vigorous stirring for 15 min. After the addition of EtOAc (60 mL), the phases were separated, and the organic phase was washed with saturated brine (60 mL), and dried over anhydrous Na_2_SO_4_. The solvent was evaporated, and the crude product was purified by column chromatography (CH_2_Cl_2_/MeOH = 20/1 (*v*/*v*)).

##### General Procedure G: Carbamate Synthesis with Phenyl Isocyanate

The phenol derivative (1.0 equiv.) was dissolved in anhydrous CH_2_Cl_2_ under Ar. 4-dimethylaminopyridine (DMAP) (1.0 equiv.), Et_3_N (1.0 equiv.), and phenyl isocyanate (PhNCO) (1.2 equiv.) were added subsequently under Ar, and the reaction mixture was stirred overnight at the room temperature. The solvent was evaporated, and the crude product was purified by column chromatography (CH_2_Cl_2_/MeOH = 20/1 (*v*/*v*)).

##### General Procedure H: Carbamate Synthesis with *N*-ethyl-*N*-methylcarbamoyl chloride

The phenol derivative (1.0 equiv.) was dissolved in pyridine (5 mL) and *N*-ethyl-*N*-methylcarbamoyl chloride (1.5 equiv.) was added. The reaction mixture was stirred for 1.5 h at room temperature. EtOAc (70 mL) and water (30 mL) were added and the resulting mixture was transferred into a separating funnel. Aqueous phase was additionally extracted with EtOAc (70 mL). Combined organic phases were washed with saturated brine (50 mL) and dried over anhydrous Na_2_SO_4_. The solvent was evaporated, and the crude product was purified by column chromatography (CH_2_Cl_2_/MeOH = 20/1]) and reversed-phase chromatography (Isolera Biotage, SNAP Biotage KP-C18-HS column, 30 g; mobile phase A (0.1% TFA (*v*/*v*) in water), B (MeCN) and C (MeOH); gradient 0−2 min, 100% A; 2−12 min, 100−0% A and 0−100% B; 12−18 min, 100% C; flow: 20 mL/min). Pure fractions were collected and organic solvent was evaporated. Aqueous phase was basified to pH = 12 (1 M NaOH_(aq)_) and extracted with CH_2_Cl_2_ (2 × 50 mL). The combined organic phases were washed with saturated brine (40 mL), dried over anhydrous Na_2_SO_4_, and the solvent was evaporated.

#### 3.1.3. Synthetic and Analytical Data for Intermediates and Inhibitors

*tert-Butyl 4-formylpiperidine-1-carboxylate***(1)** was synthesized as described in [29].

##### *tert-Butyl (E/Z)-4-(4-methoxystyryl)piperidine-1-carboxylate* (isomer (E)) (**2**)

Synthesized form (4-methoxybenzyl)triphenylphosphonium chloride (1.1 equiv., 10.60 g, 25.3 mmol), aldehyde **1** (1.0 equiv., 4.91 g, 23.0 mmol), and NaHMDS solution in THF (38%, ca. 1.9 mol/L, 14.5 mL, 27.6 mmol) via general procedure B. Overall yield of reaction: 60% (4.4 g); isolated pure *E* isomer, 3.1 g; mixture of *E* and *Z* isomers, 1.3 g. **2** (isomer (*E*)): R*_f_* = 0.18 (EtOAc/*n*-hexane = 1/9 (*v*/*v*)); yellow oil. ^1^H-NMR (400 MHz, CDCl_3_): *δ* (ppm) = 7.30–7.26 (m, 2H), 6.86–6.82 (m, 2H), 6.33 (d, *J* = 15.6 Hz, 1H), 6.00 (dd, *J* = 16.0, 6.9 Hz, 1H), 4.12 (m, 2H), 3.82–3.78 (m, 3H), 2.77 (t, *J* = 11.5 Hz, 2H), 2.30–2.21 (m, 1H), 1.77–1.71 (m, 2H), 1.46 (s, 9H), 1.43–1.28 (m, 2H).

##### *tert-Butyl (E/Z)-4-(3-methoxystyryl)piperidine-1-carboxylate* (isomer (E)) (**3**)

Synthesized form (3-methoxybenzyl)triphenylphosphonium chloride (1.1 equiv., 5.49 g, 13.1 mmol), aldehyde **1** (1.0 equiv., 2.56 g, 12.0 mmol), and NaHMDS (2 M solution in THF, 1.2 equiv., 7.6 mL, 14.4 mmol) via general procedure B. Overall yield of reaction: 63% (2.4 g); isolated pure *E* isomer, 1.2 g; mixture of *E* and *Z* isomers, 1.2 g. **3** (isomer (*E*)): R*_f_* = 0.36 (EtOAc/*n*-hexane = 1/9 (*v*/*v*)); yellow oil. ^1^H-NMR (400 MHz, CDCl_3_): *δ* (ppm) = 7.22 (m, 1H), 6.94 (d, *J* = 7.7 Hz, 1H), 6.89–6.87 (m, 1H), 6.76 (ddd, *J* = 8.2, 2.5, 0.7 Hz, 1H), 6.36 (d, *J* = 16.0 Hz, 1H), 6.14 (dd, *J* = 16.0, 6.9 Hz, 1H), 4.13 (bs, 2H), 3.81 (s, 3H), 2.78 (t, *J* = 12.0 Hz, 2H), 2.33–2.22 (m, 1H), 1.73–1.67 (m, 2H), 1.47 (s, 9H), 1.14 (dt, *J* = 12.3, 4.1 Hz, 1H), 1.11 (dt, *J* = 12.3, 4.1 Hz, 1H).

##### *tert-Butyl 4-(4-methoxyphenethyl)piperidine-1-carboxylate* (**4**)

Synthesized form *tert*-butyl (*E*/*Z*)-4-(4-methoxystyryl)piperidine-1-carboxylate (1.0 equiv., 1.30 g, 4.1 mmol) via general procedure C. Yield: quantitative (1.3 g); R*_f_* = 0.19 (EtOAc/*n*-hexane = 1/9 (*v*/*v*)); colorless oil. ^1^H-NMR (400 MHz, CDCl_3_): *δ* (ppm) = 1.12 (ddd, *J* = 16.3, 12.5, 4.3 Hz, 2H), 1.35–1.43 (m, 1H), 1.45 (s, 9H), 1.50–1.56 (m, 2H), 1.64–1.71 (m, 2H), 2.55–2.59 (m, 2H), 2.66 (t, *J* = 11.8 Hz, 2H), 3.79 (s, 3H), 4.07 (bs, 2H), 6.81–6.84 (m, 2H), 7.07–7.10 (m, 2H).

##### *tert-Butyl 4-(3-methoxyphenethyl)piperidine-1-carboxylate* (**5**)

Synthesized form *tert*-butyl (*E*/*Z*)-4-(3-methoxystyryl)piperidine-1-carboxylate (1.0 equiv., 1.21 g, 3.8 mmol) via general procedure C. Yield: quantitative (1.2 g); R*_f_* = 0.37 (EtOAc/*n*-hexane = 1/9 (*v*/*v*)); colorless oil. ^1^H-NMR (400 MHz, CDCl_3_): *δ* (ppm) = 7.22–7.17 (m, 1H); 6.77–6.72 (m, 3H), 4.08 (bs, 2H), 3.80 (s, 3H), 2.70–2.59 (m, 4H), 1.73–1.67 (m, 2H), 1.59–1.53 (m, 2H), 1.45 (s, 9H), 1.44–1.37 (m, 1H), 1.14 (dt, *J* = 12.3, 4.1 Hz, 1H), 1.10 (dt, *J* = 12.3, 4.1 Hz, 1H).

##### *(E)-4-(4-methoxystyryl)-1-(prop-2-yn-1-yl)piperidine* (**6**)

Synthesized from **2** (1.0 equiv., 1.78 g, 5.6 mmol) via general procedures D and E. Yield: 24%; R*_f_* = 0.28 (EtOAc/*n*-hexane = 1/1 (*v*/*v*)); white crystals, mp 59–63 °C. ^1^H-NMR (400 MHz, CDCl_3_): *δ* (ppm) = 7.30–7.26 (m, 2H), 6.85–6.81 (m, 2H), 6.32 (d, *J* = 15.6 Hz, 1H), 6.02 (dd, *J* = 15.9, 7.0 Hz, 1H), 3.80 (s, 3H), 3.32 (d, J = 2.4 Hz, 2H), 2.96–2.90 (m, 2H), 2.30–2.23 (m, 2H), 2.26 (t, *J* = 2.4 Hz, 2H), 2.15–2.05 (m, 1H), 1.83–1.76 (m, 2H), 1.61–1.48 (m, 2H). ^13^C-NMR (100 MHz, CDCl_3_): *δ* (ppm) = 158.90; 132.98; 130.61; 127.70; 127.22; 114.06; 79.29; 73.07; 55.43; 52.47; 47.42; 38.96; 32.32. HRMS (ESI+): *m*/*z* calculated for C_17_H_22_NO [M + H]^+^: 256.1696; found 256.1685. HPLC purity, 100.0% (*t_R_* = 5.56 min).

##### *4-(4-methoxyphenethyl)-1-(prop-2-yn-1-yl)piperidine* (**7**)

Synthesized from **4** (1.0 equiv., 1.31 g, 4.1 mmol) via general procedures D and E. Yield: 70%; R*_f_* = 0.26 (EtOAc/*n*-hexane = 1/1 (*v*/*v*)); white crystals, mp 30–31 °C. ^1^H-NMR (400 MHz, CDCl_3_): *δ* (ppm) = 7.09–7.05 (m, 2H), 6.82–6.77 (m, 2H), 3.74 (s, 3H), 3.26 (d, *J* = 2.4 Hz, 2H), 2.88–2.83 (m, 2H), 2.58–2.54 (m, 2H), 2.22 (t, *J* = 2.4 Hz, 1H), 2.19–2.13 (m, 2H), 1.77–1.70 (m, 2H), 1.56–1.50 (m, 2H), 1.35–1.22 (m, 3H). ^13^C-NMR (100 MHz, CDCl_3_): *δ* (ppm) = 157.56; 134.58; 129.05; 113.64; 79.17; 72.86; 55.07; 52.46; 47.14; 38.47; 34.61; 32.17; 32.04. HRMS (ESI+): *m*/*z* calculated for C_17_H_24_NO [M + H]^+^: 258.1852; found 258.1843. HPLC purity, 100.0% (*t_R_* = 5.87 min).

##### *4-(3-methoxyphenethyl)-1-(prop-2-yn-1-yl)piperidine* (**8**)

Synthesized from **5** (1.0 equiv., 1.21 g, 3.8 mmol) via general procedures D and E. Yield: 61%; R*_f_* = 0.26 (EtOAc/*n*-hexane = 1/1 (*v*/*v*)); colorless oil. ^1^H-NMR (400 MHz, CDCl_3_): *δ* (ppm) = 7.21–7.16 (m, 1H), 6.79–6.70 (m, 3H), 3.79 (s, 3H), 3.29 (d, *J* = 2.4 Hz, 2H), 2.90–2.86 (m, 2H), 2.63–2.57 (m, 2H), 2.23 (t, *J* = 2.4 Hz, 1H), 2.20–2.14 (m, 2H), 1.78–1.75 (m, 2H), 1.59–1.54 (m, 2H), 1.37–1.24 (m, 3H). ^13^C-NMR (100 MHz, CDCl_3_): *δ* (ppm) = 159.73; 144.49; 129.37; 120.88; 114.28; 110.95; 79.36; 72.97; 55.25; 52.68; 47.35; 38.28; 34.89; 33.24; 32.36. HRMS (ESI+): *m*/*z* calculated for C_17_H_24_NO [M + H]^+^: 258.1852, found 258.1843. HPLC purity, 97.4% (*t_R_* = 5.69 min).

##### *(E)-4-(2-(1-(prop-2-yn-1-yl)piperidin-4-yl)vinyl)phenol* (**9**)

Synthesized from **6** (1.0 equiv., 0.33 g, 1.3 mmol) via general procedure F. Yield: 13%; R*_f_* = 0.20 (CH_2_Cl_2_/MeOH = 20/1 (*v*/*v*)); white crystals, mp 50–54 °C. ^1^H-NMR (400 MHz, CDCl_3_): *δ* (ppm) = 7.22–7.19 (m, 2H), 6.76–6.72 (m, 2H), 6.29 (d, *J* = 15.9 Hz, 1H), 5.97 (dd, *J* = 15.9, 7.0 Hz, 1H), 3.31 (d, *J* = 2.4 Hz, 2H), 3.00–2.92 (m, 2H), 2.34 (dt, *J* = 11.7, 2.2 Hz, 2H), 2.27 (t, *J* = 2.4 Hz, 2H), 2.15–2.07 (m, 1H), 1.83–1.77 (m, 2H), 1.63–1.53 (m, 2H); resonance for OH missing. ^13^C-NMR (100 MHz, CDCl_3_): *δ* (ppm) = 155.10; 132.77; 130.57; 127.76; 127.44; 115.61; 79.05; 73.31; 52.42; 47.36; 38.88; 32.18. HRMS (ESI+): *m*/*z* calculated for C_16_H_20_NO [M + H]^+^: 242,1539, found 242,1531. HPLC purity, 100.0% (*t_R_* = 3.86 min).

##### *4-(2-(1-(prop-2-yn-1-yl)piperidin-4-yl)ethyl)phenol* (**10**)

Synthesized from **7** (1.0 equiv., 0.72 g, 2.8 mmol) via general procedure F. Yield: 30%; R*_f_* = 0.18 (CH_2_Cl_2_/MeOH = 20/1 (*v*/*v*)); white crystals, mp 113–115 °C. ^1^H-NMR (400 MHz, CDCl_3_): *δ* (ppm) = 7.33 (bs, 1H), 6.98 (d, *J* = 8.1 Hz, 2H), 6.72 (d, *J* = 8.2 Hz, 2H), 3.31 (d, *J* = 2.4 Hz, 2H), 2.95–2.90 (m, 2H), 2.55–2.47 (m, 2H), 2.30–2.23 (m, 3H), 1.77–1.73 (m, 2H), 1.52–1.47 (m, 2H), 1.39–1.26 (m, 3H). ^13^C-NMR (100 MHz, CDCl_3_): *δ* (ppm) = 154.49; 133.85; 129.30; 115.65; 78.33; 73.91; 52.39; 47.02; 38.36; 34.55; 32.16; 31.68. HRMS (ESI+): *m*/*z* calculated for C_16_H_22_NO [M + H]^+^: 244.1696, found 244.1687. HPLC purity, 97.4% (*t_R_* = 5.10 min).

##### *3-(2-(1-(prop-2-yn-1-yl)piperidin-4-yl)ethyl)phenol* (**11**)

Synthesized from **8** (1.0 equiv., 0.57 g, 2.2 mmol) via general procedure F. Yield: 30%; R*_f_* = 0.18 (CH_2_Cl_2_/MeOH = 20/1 (*v*/*v*)); white crystals, mp 105–108 °C. ^1^H-NMR (400 MHz, CDCl_3_): *δ* (ppm) = 7.15–7.11 (m, 1H), 6.72 (d, *J* = 7.5 Hz, 1H), 6.66–6.62 (m, 2H), 3.31 (d, *J* = 2.4 Hz, 2H), 2.93–2.88 (m, 2H), 2.60–2.54 (m, 2H), 2.24 (t, *J* = 2.4 Hz, 1H), 2.23–2.17 (m, 2H), 1.79–1.74 (m, 2H), 1.58–1.52 (m, 2H), 1.39–1.24 (m, 3H); resonance for OH missing. ^13^C-NMR (100 MHz, CDCl_3_): *δ* (ppm) = 156.04; 144.70; 129.59; 120.70; 115.50; 112.89; 79.00; 73.36; 52.60; 47.28; 38.16; 34.82; 33.10; 32.16. HRMS (ESI+): *m*/*z* calculated for C_16_H_22_NO [M + H]^+^: 244.1696, found 244.1685. HPLC purity, 100.0% (*t_R_* = 4.24 min).

##### *4-(2-(1-(prop-2-yn-1-yl)piperidin-4-yl)ethyl)phenyl phenylcarbamate* (**12**)

Synthesized from **10** (1.0 equiv., 0.049 g, 0.2 mmol) via general procedure G. Yield: 30%; R*_f_* = 0.23 (CH_2_Cl_2_/MeOH = 20/1 (*v*/*v*)); white crystals, mp 122–125 °C. ^1^H-NMR (400 MHz, CDCl_3_): *δ* (ppm) = 7.46–7.42 (m, 2H), 7.36–7.30 (m, 2H), 7.21–7.16 (m, 2H), 7.12–7.07 (m, 3H), 6.96 (bs, 1H), 3.29 (d, *J* = 2.4 Hz, 2H), 2.92–2.87 (m, 2H), 2.65–2.61 (m, 2H), 2.22 (t, *J* = 2.4 Hz, 1H), 2.21–2.15 (m, 2H), 1.79–1.75 (m, 2H), 1.59–1.54 (m, 2H), 1.37–1.26 (m, 3H). ^13^C-NMR (100 MHz, CDCl_3_): *δ* (ppm) = 152.05; 148.62; 140.25; 137.57; 129.31; 129.26; 123.98; 121.54; 118.85; 79.30; 73.06; 52.66; 47.34; 38.36; 34.76; 32.55; 32.31. HRMS (ESI+): *m*/*z* calculated for C_23_H_27_N_2_O_2_ [M + H]^+^: 363.2067, found 363.2052. HPLC purity, 98.6% (*t_R_* = 6.94 min).

##### *3-(2-(1-(prop-2-yn-1-yl)piperidin-4-yl)ethyl)phenyl phenylcarbamate* (**13**)

Synthesized from **11** (1.0 equiv., 0.097 g, 0.4 mmol) via general procedure G. Yield: 61%; R*_f_* = 0.24 (CH_2_Cl_2_/MeOH = 20/1 (*v*/*v*)); yellow oil. ^1^H-NMR (400 MHz, CDCl_3_): *δ* (ppm) = 7.46–7.423 (m, 2H), 7.36–7.26 (m, 3H), 7.12–7.09 (m, 1H), 7.06–7.04 (m, 1H), 7.03–6.98 (m, 2H), 3.29 (d, *J* = 2.4 Hz, 2H), 2.91–2.87 (m, 2H), 2.66–2.62 (m, 2H), 2.23 (t, *J* = 2.4 Hz, 1H), 2.22–2.15 (m, 2H), 1.78–1.74 (m, 2H), 1.61–1.55 (m, 2H), 1.37–1.26 (m, 3H); resonance for NH missing. ^13^C-NMR (100 MHz, CDCl_3_): *δ* (ppm) = 150,70; 144,58; 137,55; 129,34; 129,30; 125,89; 124,04; 121,59; 119,01; 118.88; 79.30; 73.07; 52.67; 47.34; 38.09; 34.96; 33.03; 32.30. HRMS (ESI+): *m*/*z* calculated for C_23_H_27_N_2_O_2_ [M + H]^+^: 363.2067, found 363.2047. HPLC purity, 97.8% (*t_R_* = 6.63 min).

##### *4-(2-(1-(prop-2-yn-1-yl)piperidin-4-yl)ethyl)phenyl ethyl(methyl)carbamate* (**14**)

Synthesized from **10** (1.0 equiv., 0.146 g, 0.6 mmol) via general procedure H. Yield: 83%; R*_f_* = 0.24 (CH_2_Cl_2_/MeOH = 20/1 (*v*/*v*)); white crystals, mp 61–62 °C. ^1^H-NMR (400 MHz, CDCl_3_): *δ* (ppm) = 7.16–7.12 (m, 2H), 7.01 (d, *J* = 8.0 Hz, 2H), 3.50–3.36 (m, 2H), 3.28 (d, *J* = 2.4 Hz, 2H), 3.02 (s + s, 3H), 2.90–2.85 (m, 2H), 2.64–2.57 (m, 2H), 2.22 (t, *J* = 2.4 Hz, 1H), 2.20–2.12 (m, 2H), 1.77–1.73 (m, 2H), 1.58–1.52 (m, 2H), 1.36–1.15 (m, 6H). ^13^C-NMR (100 MHz, CDCl_3_): *δ* (ppm) = 154.90; 149.59; 139.51; 129.10; 121.61; 79.35; 72.95; 52.65; 47.34; 44.14; 38.34; 34.67; 34.32; 33.88; 32.48; 32.33. HRMS (ESI+): *m*/*z* calculated for C_20_H_29_N_2_O_2_ [M + H]^+^: 329.2224, found 329.2209. HPLC purity, 100.0% (*t_R_* = 6.13 min).

##### *3-(2-(1-(prop-2-yn-1-yl)piperidin-4-yl)ethyl)phenyl ethyl(methyl)carbamate* (**15**)

Synthesized from **11** (1.0 equiv., 0.049 g, 0.2 mmol) via general procedure H. Yield: 38%; R*_f_* = 0.28 (CH_2_Cl_2_/MeOH = 20/1 (*v*/*v*)); yellow oil. ^1^H-NMR (400 MHz, CDCl_3_): *δ* (ppm) = 7.26–7.23 (m, 1H), 6.99 (d, *J* = 7.7 Hz, 1H), 6.95–6.90 (m, 2H), 3.50–3.37 (m, 2H), 3.29 (d, *J* = 2.4 Hz, 2H), 3.02 (s + s, 3H), 2.91–2.86 (m, 2H), 2.64–2.60 (m, 2H), 2.23 (t, *J* = 2.4 Hz, 1H), 2.21–2.14 (m, 2H), 1.78–1.74 (m, 2H), 1.60–1.53 (m, 2H), 1.34–1.17 (m, 6H). ^13^C-NMR (100 MHz, CDCl_3_): *δ* (ppm) = 154.81; 151.64; 144.26; 129.09; 125.26; 121.67; 119.11; 79.34; 72.97; 52.66; 47.33; 44.15; 38.11; 34.98; 34.33; 33.89; 33.02; 32.30. HRMS (ESI+): *m*/*z* calculated for C_20_H_29_N_2_O_2_ [M + H]^+^: 329.2226, found 329.2218. HPLC purity, 96.5% (*t_R_* = 2.42 min).

### 3.2. Biological Evaluation

#### 3.2.1. In Vitro Cholinesterase Assay

The inhibitory potencies of the compounds against the ChEs were determined using the Ellman method [31,32]. 5,5′-Dithiobis (2-nitrobenzoic acid) (Ellman’s reagent; DTNB, St. Louis, MO, USA), butyrylthiocholine iodide, and acetylthiocholine iodides were from Sigma-Aldrich. Recombinant hAChE and hBChE at the stock concentration of 16.5 mg/mL and 14.5 mg/mL, respectively, were kindly donated by Xavier Brazzolotto, Florian Nachon, and José Dias (IRBA, Brétigny-sur-Orge, France). The enzyme solutions were prepared by dilution of the concentrated stocks in 0.1 M phosphate-buffered solution (pH 8.0). The reactions were carried out in a final volume of 300 µL of 0.1 M phosphate-buffered solution, pH 8.0, containing 370 µM DTNB, 500 μM butyrylthiocholine/acetylthiocholine, and approximately 1 nM or 50 pM hBChE or hAChE, respectively. The reactions were started by the addition of the substrate after 5-min pre-incubation at room temperature. For time-dependency measurements of hBChE inhibition, the pre-incubation time was varied (i.e., 1 min, 5 min, 15 min, 30 min). The final content of the organic solvent (DMSO) was always 1%. The formation of the yellow 5-thio-2-nitrobenzoate anion as a result of the reaction of DTNB with the thiocholines was monitored for 1 min as the change in absorbance at 412 nm, using a 96-well microplate reader (SynergyTM H4; BioTek Instruments, Inc., USA). The initial velocities in the presence (v_i_) and the absence (v_o_) of the test compounds were calculated. The inhibitory potencies were expressed as the residual activities, according to RA = (v_i_ − *b*)/(v_o_ − *b*), where *b* is the blank value using phosphate buffer without ChEs. For the IC_50_ measurements, seven different concentrations of each compound were used to obtain enzyme activities of between 5% and 90%. The IC_50_ values were obtained by plotting the residual ChEs activities against the applied inhibitor concentrations, with the experimental data fitted to a four-parameter logistic function (GraphPad Prism 9.0; GraphPad Software Inc., San Diego, CA, USA).

#### 3.2.2. In Vitro MAO-A/B Assay

The effects of the test compounds on hMAO were investigated using a fluorimetric assay [32,33]. Recombinant hMAO, expressed in BTI-TN-5B1-4 insect cells, *p*-tyramine hydrochloride, and horse-radish peroxidase type II were purchased from Sigma Aldrich, whereas Amplex Red was synthesized as reported previously [36].

In brief, 100 µL 50 mM sodium phosphate buffer (pH 7.4, 0.05 vol.% Triton X-114) containing the compounds or the reference inhibitors and hMAO were incubated for 15 min at 37 °C. The reaction was started by adding Amplex Red (final concentration, 250 µM), horseradish peroxidase (final activity, 2 U/mL), and *p*-tyramine (final concentration, 1 mM). The fluorescence increase (λ_ex_ = 530 nm, λ_em_ = 590 nm) at 37 °C was monitored for 20 min. DMSO was used for control experiments. To determine the blank value (b), phosphate-buffered solution replaced the enzyme. Each measurement was performed in duplicate. The inhibitory potencies were expressed as the residual activities (RA) and IC_50_ values as described in Section 3.2.1. For the reversibility assay, the previously described protocol was followed [32].

#### 3.2.3. Molecular Docking

The computations were performed on a Lenovo IdeaPad L340-17IRH Gaming Laptop with six dual-core Intel i7-9750H 2.6 GHz processors, 16 GB RAM, 1 TB SSD hard drive, running Windows 10 Home, release 10.0.19041. Virtual screening was performed with Schrödinger Suite Release 2018-1 (Schrödinger, LLC, New York, USA, 2018). Ligand structures were prepared with LigPrep and ionized with Epik (pH 7 ± 2) using OPLS3 force field [37]. The crystal structures were obtained from PDB and prepared with Protein Preparation Wizard.

For hBChE, the best resolution (1.9 Å) crystal structure 6QAA was used [38]. Chain A with the co-crystallized ligand was retained while other ligands and water molecules were removed. Hydrogen bonds were assigned using PROPKA (pH 7.0) and the structure was minimized. A receptor grid was then generated with van der Waals radii scaling by 1, partial charge cutoff was set to 0.25, and halogens were enabled as donors. The active site was defined as the centroid of the co-crystallized ligand with innerbox size 10 Å^3^ and outerbox size 24.938 Å^3^. The rotation of hydroxyls for Ser198, Glu197, and Tyr332 was enabled. Docking was performed using Glide XP with default settings [39]. The highest scoring poses were visualized with Maestro. Redocking of two cognate ligands (PDB 6QAE–RMSD 2.5052 Å, PDB 6SAM–RMSD 1.1887 Å) was used to validate the model.

The CovDock protocol was used for docking of the carbamate ligands [40]. Ser198 was chosen as the reactive residue, the box was defined as the centroid of the co-crystallized ligand with size defined by the ligand. Reaction type was a nucleophilic addition to a double bond, producing a tetrahedral intermediate. No constraints were imposed and the thorough pose prediction docking mode was used.

For hMAO-B, the chain A of 6RKP crystal structure (1.7 Å resolution) [41] was used and processed as described above. The CovDock protocol was used for docking of the propargyl ligands [40]. The FAD cofactor, A:600, was chosen as the reactive residue, the box was defined as the centroid of the co-crystallized ligand with innerbox size 10 Å^3^ and outerbox size 30 Å^3^. The reaction was encoded using a custom chemistry file (LIGAND_SMARTS_PATTERN 1,C#CCNC; RECEPTOR_SMARTS_PATTERN 13,O=c1[nH]c(=O)[nH]c(c12)N(C)c3c(N2)cc(C)c(C)c3; CUSTOM_CHEMISTRY (“<1>|<2>”, (“bond”,1,(1,2))); CUSTOM_CHEMISTRY (“<2>#C”, (“bond”,1,(1,2)))), which resulted in a single bond between *N*^5^ of the FAD cofactor and terminal propargylic carbon of the ligand. Due to discrepancies between different crystal structures, the covalently bound propargyls were uniformly modelled as *sp*^3^-hybridized. No constraints were imposed and the thorough pose prediction docking mode was used with an initial GScore cutoff of 2.5 kcal/mol and 200 initial poses, as per default settings. The docking protocol was validated by comparing the highest CovDock-scored pose with the cognate ligand pose (Table 2).

#### 3.2.4. Statistical Analysis

The reversibility was analyzed by one-way ANOVA and post-hoc comparisons were made using two-tailed Student’s *t*-test. *p* < 0.05—statistically significant. All data are means ± SEM (*n* = 2).

## 4. Conclusions

To conclude, we synthesized dual hBChE/hMAO-B inhibitors **13**–**15**, among which derivative **15** exhibited balanced, low micromolar inhibition of both enzymes (IC_50_ (hBChE) = 8.5 ± 0.9 µM, IC_50_ (hMAO-B) = 4.3 ± 0.8 µM). As anticipated, the carbamate moiety resulted in covalent hBChE inhibition, as supported by the docking studies and time-dependent inhibition of the enzyme. Therefore, the compound **15** represents an excellent candidate for further development of an efficient MTDL. Nonetheless, future cellular and in vivo studies in animal models of AD will reveal the therapeutic potential of this compound.

## Figures and Tables

**Table 1:** Inhibitory potencies and structures of synthesized analogues. Compounds with RA above 50% were considered inactive. IC_50_ values are presented as means ± S.E.M of 3 independent experiments.

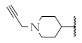		RA ± SD (%) at 100 µM or IC_50_ ± SEM			
Compound	Substituent	hAChE	hBChE	hMAO-A	hMAO-B
**6**	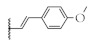	61.9 ± 1.2%	68.8 ± 0.6%	52.6 ± 0.7%	**72.3 ± 7.5 nM**
**7**	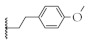	73.1 ± 4.3%	51.2 ± 1.6%	62.2 ± 1.8%	**93.8 ± 4.1 nM**
**8**	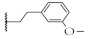	73.2 ± 8.9%	55.5 ± 0.2%	59.8 ± 3.7%	**238.4 ± 26.9 nM**
**9**	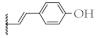	65.9 ± 3.1%	64.9 ± 2.0%	**15.5 ± 0.9 µM**	**12.9 ± 1.1 µM**
**10**	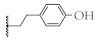	61.4 ± 2.7%	53.8 ± 0.6%	**34.0 ± 5.8 µM**	**12.6 ± 2.8 µM**
**11**	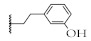	54.7 ± 2.9%	72.9 ± 2.8%	63.3 ± 0.4%	**52.5 ± 14.8 µM**
**12**	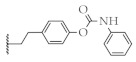	52.2 ± 1.7%	60.2 ± 1.8%	56.7 ± 0.9%	**9.7 ± 1.2 µM**
**13**	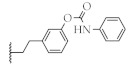	51.9 ± 1.4%	**4.4 ± 0.8 µM**	54.0 ± 0.4%	**23.6 ± 3.0 µM**
**14**	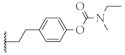	66.1 ± 4.8%	**75.5 ± 8.4 µM**	51.2 ± 1.5%	**181.4 ± 28.7 nM**
**15**	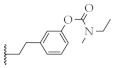	60.2 ± 1.8%	**4.3 ± 0.8 µM**	69.4 ± 9.4%	**8.5 ± 0.9 µM**

**Table 2:** hMAO-B covalent docking validation.

PDB Crystal Structure	RMSD of Highest CovDock-Scored vs. Cognate Pose
1S2Q	3.3049
1S2Y	2.4053
1S3B	1.7822
1S3E	1.9623
2BYB	0.9706
2C65	1.2612
2C66	1.5559
4CRT	1.0416
5MRL	1.6187
6RKB	0.5906
6RKP	1.0418
6RLE	0.7104

## Data Availability

Not applicable.

## Sample Availability

Samples of the compounds are available from the authors.
